# Progression of early diagnostic markers for diabetic kidney disease: From single-indicator detection to multi-omics integration modeling

**DOI:** 10.1016/j.crphys.2026.100190

**Published:** 2026-08-11

**Authors:** Jiao Meng, Wei Zhang, Hanmin Wang, Zihan He, Zhuxian Zhang

**Affiliations:** Department of Endocrinology and Metabolism, Qujing Central Hospital of Yunnan Province (Qujing First People's Hospital), No. 819 Shengfeng Road, Xicheng Sub-district, Qilin District, Qujing City, 655000, PR China

**Keywords:** Diabetic kidney disease, Early diagnosis, Biomarkers, Metabolomics, Machine learning

## Abstract

Diabetic kidney disease (DKD) is the leading cause of end-stage renal disease (ESRD), but early diagnosis remains challenging. Current reliance on indicators such as urinary albumin and glomerular filtration rate is limited by insufficient sensitivity and susceptibility to interference. The pathogenesis of DKD is complex, involving multiple intersecting pathways and epigenetic modifications in the progression of the disease. Omics biomarkers offer new directions for early risk assessment, while machine learning can integrate multi-omics data to build efficient diagnostic models. This article reviews recent advances in biomarker research, discusses strategies for model construction, and provides theoretical references for early and precise diagnosis and clinical translation of DKD.

## Highlights

• The pathogenesis of diabetic kidney disease is complex, involving multiple intersecting pathways and epigenetic modifications in the progression of the disease.
• Omics biomarkers offer new directions for early risk assessment of diabetic nephropathy, and machine learning can integrate multi-omics data to build efficient diagnostic models.
• Traditional indicators for diagnosing diabetic kidney disease have limitations: an elevated UACR often lags behind early kidney damage, some DKD patients may present with ‘non-albuminuric kidney disease,’ and the sensitivity of eGFR is limited.

DKD is the most common microvascular complication of diabetes mellitus (DM) and the leading cause of end-stage renal disease (ESRD). Early diagnosis and intervention are crucial for slowing its progression. Currently, clinical practice relies on the urine albumin-to-creatinine ratio (UACR) and estimated glomerular filtration rate (eGFR) as the core diagnostic indicators. However, both have limitations in identifying early DKD, and there is also a normoalbuminuric type of DKD. Previous studies have focused on the estimated glomerular filtration rate (eGFR) based on creatinine (eGFRcr), cystatin C (eGFRcys), and their combination (eGFRcr-cys), exploring their diagnostic value for early renal injury in DM patients. Nonetheless, these indicators still have inherent diagnostic delays in clinical application, making it difficult to meet the needs of early intervention (Zhang et al., 2021). Renal biopsy, as the diagnostic gold standard, is limited in use due to its invasiveness. The pathogenesis of DKD is complex, involving interactions among multiple pathways such as hyperglycemia, hemodynamic abnormalities, inflammation, oxidative stress, and fibrosis, eventually leading to progressive structural damage to the kidneys. Epigenetic modifications may explain why damage continues to progress even after glycemic control. Therefore, surpassing traditional indicators and identifying earlier biomarkers has become a research focus. Genomics and metabolomics analyses offer new directions for early risk assessment. At the same time, machine learning techniques can effectively integrate multi-omics data to construct early diagnostic models with high sensitivity and specificity. This article aims to systematically review research progress on related biomarkers, and to discuss strategies and challenges in multi-omics integration and machine learning modeling, in hopes of providing a reference for precise early diagnosis and clinical translation of DKD.

## 1 Single biomarkers

### 1.1 Biomarkers reflecting glomerular injury and filtration barrier dysfunction

Podocyte injury is a key early event in DKD, and increased excretion of podocyte-specific proteins in the urine can directly reflect damage to the slit diaphragm. Among them, urinary Nephrin levels increase with disease progression, with a diagnostic sensitivity of 100% and a specificity of 88% (Veluri and Mannangatti, 2022); Podocin mRNA in urine sediment increases as early as in the initial stages, and its ability to predict a decline in eGFR is comparable to that of urinary albumin (Fukuda et al., 2020); the urinary excretion of Podocalyxin is already significantly increased during the stage when urinary albumin levels are normal (Zeng et al., 2023). These markers make it possible to identify podocyte injury in the preclinical stage.

Thickening of the glomerular basement membrane (GBM) is a core early pathological feature of DKD, arising from an imbalance in the metabolism of the basement membrane and extracellular matrix (ECM) components (Adeva-Andany and Carneiro-Freire, 2022). Type IV collagen is the main scaffold protein of the GBM, and its urinary excretion increases even when urinary albumin levels are still normal, being associated with subsequent declines in eGFR (Katavetin et al., 2010). Elevated urinary laminin levels may precede the appearance of microalbuminuria (Yurchenco and Kulczyk, 2024). Damage to the basement membrane also leads to abnormal filtration of serum proteins. Transferrin (TRF) is extremely sensitive to damage to the charge barrier, and its urinary excretion can rise earlier than microalbuminuria (Kamel et al., 2022). Urinary excretion of immunoglobulin G (IgG) indicates structural barrier damage and shows good diagnostic performance even at the microalbuminuria stage (Abdou et al., 2022). The urinary excretion of ceruloplasmin (Cp) is also a signal of early damage to the charge barrier, and may participate in disease progression by interfering with local metal metabolism (Jiayi et al., 2024). These biomarkers (Type IV collagen, laminin, etc., which directly reflect ECM remodeling; TRF, IgG, Cp, etc., which reflect loss of filtration function) change even before abnormalities in traditional urinary albumin indicators, providing important evidence for the early identification of DKD.

Glomerular endothelial cell injury and activation are the key initial events in DKD microvascular lesions. Among related biomarkers, the upregulation of vascular endothelial growth factor (VEGF-A) can disrupt endothelial junctions and directly drive proteinuria (Nussdorfer et al., 2024); von Willebrand factor (vWF) increases as early as the microalbuminuria stage, which may be disease-specific and can promote microthrombosis formation (Ono et al., 2019); elevated levels of soluble adhesion molecules (sICAM-1/sVCAM-1) reflect an inflammatory state of the endothelium (Rania et al., 2024). These biomarkers reveal the mechanisms of endothelial injury from different perspectives and have potential for early diagnosis.

### 1.2 Biomarkers reflecting tubular injury and dysfunction

Biomarkers indicating damage to renal tubular epithelial cells are crucial for the early diagnosis of DKD. Neutrophil gelatinase-associated lipocalin (NGAL) rises rapidly after kidney injury; its urinary levels increase with the progression of DKD, showing a positive correlation with UACR and a negative correlation with eGFR (Swaminathan et al., 2025). Kidney injury molecule-1 (KIM-1) is specifically upregulated during tubular injury; its baseline levels can serve as an early warning for injury, with diagnostic specificity as high as 90% (Varatharajan et al., 2024). Liver-type fatty acid-binding protein (L-FABP) is associated with tubular oxidative stress. Its levels are elevated even at the normoalbuminuria stage and continue to rise as the disease progresses, correlating positively with UACR and negatively with eGFR (Ma et al., 2024).

β2-microglobulin (β2-MG) levels increase significantly in the early stages of DKD and escalate with worsening proteinuria, serving as a sensitive signal that appears before traditional markers show abnormalities (Yang et al., 2022). Retinol-binding protein 4 (RBP4) circulatory levels are markedly increased in T2DM patients with renal injury, show a positive correlation with UACR and negative correlation with eGFR, and demonstrate strong diagnostic efficacy (Cao et al., 2024). These biomarkers can reflect early tubular reabsorption dysfunction in DKD.

### 1.3 In DKD, biomarkers associated with pathological processes such as inflammation and oxidative stress are also of significant importance

Monocyte chemoattractant protein-1 (MCP-1) is a key factor in recruiting inflammatory cells; its urinary levels are markedly elevated in rapidly progressive DKD and serve as an efficient predictor of disease progression (Swaminathan et al., 2025). 8-Hydroxy-2′-deoxyguanosine (8-OHdG), a specific product of oxidative DNA damage, shows increased levels in both serum and urine of DKD patients, and is negatively correlated with renal function (Spoto et al., 2025). The soluble forms of tumor necrosis factor receptors 1 and 2 (TNFR1/TNFR2) are novel inflammatory markers; their serum levels rise before a significant decline in kidney function, with TNFR2 being significantly associated with decreased eGFR (Lousa et al., 2023).

### 1.4 Single-type biomarkers have multiple limitations in clinical application

Firstly, different studies often report contradictory findings and vary greatly in predictive efficacy. This is due both to the inherent biological characteristics of the biomarkers themselves (for example, NGAL and KIM-1 can be affected by a variety of systemic or non-specific renal injuries, leading to insufficient specificity), and to the lack of internationally standardized detection protocols, which impedes data comparability and clinical translation. Secondly, there is no clear consensus on the optimal source of biological samples. Finally, the performance of these biomarkers may vary depending on the type of DM and ethnic population, further increasing the complexity of widespread application. Table 1 systematically compares the specificity grades and evidence limitations of these biomarkers accordingly.

**Table 1:** Single biomarkers for early detection of diabetic kidney disease: Specificity status and evidence quality assessment.

Biomarker Category	Specific Biomarker	Proposed Mechanism/Reflection	Evidence in DKD	Specificity to DKD	Key Limitations/Notes	Ref
Podocyte/GlomerularFiltration Barrier Injury	Nephrin	Podocyte slit diaphragm injury; urinary excretion increases with DKD progression	Sensitivity 100%, Specificity 88% in DKD cohorts	○Limited	Also elevated in other glomerular diseases (e.g., minimal change disease, FSGS); not pathognomonic for DKD	Veluri and Mannangatti (2022)
	Podocin mRNA	Decreased podocyte expression, leakage into urine after injury	Early elevation in DKD; predictive of eGFR decline comparable to albuminuria	○Limited	Limited DKD cohort validation; may reflect general podocyte stress	Fukuda et al. (2020)
	Podocalyxin	Podocyte glycocalyx disruption; early urinary excretion before albuminuria	Elevated in normoalbuminuric stage	○Limited	Preliminary evidence; specificity for DKD unestablished	Zeng et al. (2023)
	Type IV Collagen	GBM thickening; ECM remodeling	Increased urinary excretion before albuminuria; correlates with future eGFR decline	×Poor	GBM remodeling occurs in multiple chronic kidney diseases; not DKD-specific	(Adeva-Andany and Carneiro-Freire, 2022; Katavetin et al., 2010)
	Laminin	Basement membrane and ECM component metabolism imbalance	Elevated before microalbuminuria onset	×Poor	ECM changes are common to progressive kidney diseases regardless of etiology	Yurchenco and Kulczyk (2024)
	TRF	Charge barrier dysfunction; highly sensitive to anionic barrier loss	Urinary excretion rises before microalbuminuria	×Poor	Charge barrier injury occurs in any glomerular disease (e.g., membranous nephropathy)	Kamel et al. (2022)
	IgG	Structural barrier compromise; size-selectivity loss	Good diagnostic performance in microalbuminuria stage	×Poor	IgGuria present in various proteinuric kidney diseases; not DKD-specific	Abdou et al. (2022)
	Cp	Charge barrier injury; local metal metabolism disturbance	Early signal of charge barrier damage	×Poor	Limited DKD evidence; may reflect systemic copper metabolism rather than kidney-specific injury	Jiayi et al. (2024)
Glomerular Endothelial Injury	VEGF-A	Endothelial junction disruption; drives proteinuria via permeability increase	Upregulated in DKD; directly promotes proteinuria	×Poor	VEGF-A dysregulation occurs in multiple vascular and renal pathologies (e.g., preeclampsia, cancer); not DKD-specific	Nussdorfer et al. (2024)
	vWF	Endothelial activation; microthrombosis promotion	Elevated in microalbuminuria stage; possible disease specificity suggested	△Moderate	May have DKD specificity (as noted in original text), but requires validation in larger cohorts and comparison with non-DKD diabetic patients	Ono et al. (2019)
	sICAM-1/sVCAM-1	Endothelial inflammatory activation; leukocyte adhesion	Elevated levels reflect endothelial inflammation	×Poor	Systemic inflammation markers; elevated in atherosclerosis, infection, autoimmune diseases	Rania et al. (2024)
Tubular Injury & Dysfunction	NGAL	Proximal tubular epithelial injury; rapid response to kidney damage	Increases with DKD progression; correlates with UACR and eGFR	×Poor	Classic AKI biomarker; elevated in any acute or chronic tubular injury (e.g., ischemia, toxins, obstruction)	Swaminathan et al. (2025)
	KIM-1	Tubular epithelial injury; specific upregulation in damaged tubules	Baseline levels predict early injury; specificity ∼90% for tubular damage	○Limited	Specific for tubular injury but NOT specific for DKD etiology; elevated in any cause of tubular damage	Varatharajan et al. (2024)
	L-FABP	Tubular oxidative stress; fatty acid metabolism in proximal tubule	Elevated in normoalbuminuria stage; progresses with disease	○Limited	Oxidative stress marker; may be elevated in other conditions with tubular hypoxia/oxidative stress	Ma et al. (2024)
	β2-MG	Tubular reabsorption dysfunction	Early elevation in DKD; increases with proteinuria severity	×Poor	Non-specific marker of decreased GFR and tubular dysfunction; elevated in any CKD stage	Yang et al. (2022)
	RBP4	Tubular reabsorption impairment; retinol transport dysfunction	Circulating levels elevated in T2DM with kidney injury; correlates with UACR and eGFR	Limited	May be influenced by systemic metabolic state (obesity, insulin resistance); limited DKD-specific validation	Cao et al. (2024)
Inflammation &Oxidative Stress	MCP-1	Monocyte chemotaxis; inflammatory cell recruitment to kidney	Significantly elevated in rapid-progression DKD; efficient progression predictor	×Poor	Inflammatory chemokine elevated in any chronic kidney disease and systemic inflammatory conditions	Swaminathan et al. (2025)
	8-OHdG	Oxidative DNA damage; marker of oxidative stress	Elevated in serum and urine; correlates negatively with renal function	×Poor	Systemic oxidative stress marker; elevated in diabetes, aging, cancer, and any condition with increased ROS	Spoto et al. (2025)
	TNFR1/TNFR2	Soluble forms of TNF receptors; inflammatory signaling	Elevated before significant eGFR decline; TNFR2 correlates with eGFR decline	○Limited	Emerging markers with promising data but limited large-scale DKD cohort validation; may reflect systemic inflammation	Lousa et al. (2023)

Table 1. Single Biomarkers for Early Detection of Diabetic Kidney Disease: Specificity Status and Evidence Quality Assessment. This table summarizes key biomarkers for early DKD detection, categorized by their site of injury, proposed mechanism, supporting evidence, specificity to DKD, and key limitations. Note that no single biomarker achieves high specificity for DKD, as most reflect general kidney injury and may be elevated in non-diabetic kidney diseases. Multimarker panels combined with clinical variables (eGFR, UACR, diabetes duration, HbA1c) are recommended to improve diagnostic specificity. **Abbreviations**: AKI, acute kidney injury; CKD, chronic kidney disease; DKD, diabetic kidney disease; ECM, extracellular matrix; FSGS, focal segmental glomerulosclerosis; GBM, glomerular basement membrane; MCD, minimal change disease; MAU, microalbuminuria; T2DM, type 2 diabetes mellitus; UACR, urinary albumin-to-creatinine ratio; eGFR, estimated glomerular filtration rate; NGAL, neutrophil gelatinase-associated lipocalin; KIM-1, kidney injury molecule-1; L-FABP, liver-type fatty acid-binding protein; vWF, von Willebrand factor; sICAM-1, soluble intercellular adhesion molecule-1; vCAM-1, vascular cell adhesion molecule-1; MCP-1, monocyte chemoattractant protein-1; 8-OHdG, 8-hydroxydeoxyguanosine; TNFR1/2, tumor necrosis factor receptors 1/2. **Footnotes**: * Specificity grading: ×Poor (non-specific, elevated in multiple conditions); △ Moderate (potential specificity, requires validation); ○Limited (preliminary evidence, not fully validated). † Also elevated in non-DKD kidney diseases (e.g., glomerulonephritis, AKI, hypertensive nephropathy). ‡ Limited validation in large prospective DKD cohorts; evidence primarily from cross-sectional or small studies. § Systemic confounder: marker may be influenced by non-renal conditions (inflammation, infection, metabolic syndrome).

## 2 Multi-omics integration and model construction

### 2.1 Metabolomics biomarkers

Metabolomics, through the quantitative analysis of all small-molecule metabolites (typically <1500 Da) in biological fluids, directly reflects the final biochemical phenotype of an organism under specific physiological or pathological conditions. It serves as a bridge between genotype and clinical manifestation. The metabolite changes identified by metabolomics profoundly illustrate the complex pathophysiological states during the progression of DKD, such as glomerular hypertension, inflammation, oxidative stress, and mitochondrial dysfunction. Urine, as a non-invasive sample that can directly reflect the local metabolic state of the kidneys, is widely used in this field. Studies have shown that urine metabolite profiling can not only distinguish DKD from non-diabetic kidney disease, but also reveal key molecules associated with disease progression (He et al., 2025). For example, inositol has been identified as a novel prognostic biomarker significantly associated with DKD progression (Kwon et al., 2023).

Blood samples provide systemic metabolic information. Research has found that specific plasma metabolites—including lipids, amino acids, and carbohydrates—undergo significant changes at different stages of DKD, closely related to oxidative stress, inflammation, and fibrosis (Hasegawa and Inagi, 2021). A study using LC-MS/MS technology identified several differential metabolites, including lactate, L-ornithine, and L-tryptophan, and pointed out that disruptions in pathways such as amino acid biosynthesis and arginine-proline metabolism are among the core pathological mechanisms of DKD (Chen et al., 2025).

In addition, emerging metabolomics research on extracellular vesicles (EVs) reveals another dimension of biomarkers. By analyzing plasma EVs from DKD patients at different stages, researchers have discovered stage-specific metabolomic changes and identified several stable differential metabolites, including docosahexaenoic acid (DHA) and arachidonic acid (AA). This indicates that the metabolite composition within EVs can itself serve as a novel biomarker reflecting disease status (Pan et al., 2022).

In summary, metabolomics has uncovered groups of potential biomarkers from multiple levels—urine, blood, and EVs—that can warn of DKD risk and reveal its molecular mechanisms. However, the diagnostic performance of any single metabolomic biomarker remains limited by external confounders such as diet, drugs, and gut microbiota, underscoring the need for multi-omics integration.

### 2.2 Genomics-derived biomarkers

Genomics mainly seeks biomarkers from the perspectives of genetic susceptibility and epigenetic regulation, helping to identify populations at high risk for DKD and to explain disease heterogeneity. Genome-wide association studies (GWAS) have identified multiple genetic loci associated with the risk of developing DKD in patients with T2DM. Among these, the ELMO1 gene is the most extensively studied and has been validated as a DKD susceptibility gene across different ethnic groups. It is involved in key pathological processes of DKD such as apoptosis, inflammation, and fibrosis (Azarboo et al., 2024). In addition, genetic loci such as ACACB, CARS2, APOL1, and UNC13B are also thought to be associated with DKD susceptibility (Pan et al., 2023). Although these single nucleotide polymorphisms (SNPs) alone are insufficient for direct diagnosis of DKD, they have significant potential for assessing individual genetic susceptibility.

Epigenetic modifications act as a bridge between genetics and environment, offering new perspectives for understanding the mechanisms of DKD and for discovering new biomarkers. Major epigenetic modifications include DNA methylation, histone modification, and non-coding RNAs (ncRNA), which reversibly regulate gene expression and are easily influenced by environmental factors such as high glucose levels (Gu, 2019). ncRNAs do not code for proteins, but they regulate gene expression through various mechanisms and participate in the development and progression of DKD.

miRNAs are a class of short-chain ncRNAs that regulate gene expression post-transcriptionally by binding to target messenger RNAs. In DKD, the expression of various miRNAs changes significantly, participating in the regulation of core processes such as the TGF-β signaling pathway, epithelial-mesenchymal transition (EMT), and fibrosis (Sandholm et al., 2023). miRNAs are stably present in body fluids such as serum, plasma, and urine, making them well-suited as non-invasive “liquid biopsy” biomarkers (Uil et al., 2021). Research has identified multiple miRNAs associated with DKD; for example, miR-21 is regarded as an early marker of type 1 diabetic nephropathy (Fouad et al., 2020), and its promotion of fibrosis has been confirmed in animal models of T2DM-associated nephropathy (Dhas et al., 2023). miR-192-5p is significantly downregulated in renal tissue of DKD patients, and its overexpression can alleviate hyperglycemia-induced cellular damage (Wang et al., 2023). miR-145-5p is specifically overexpressed in DKD models, and its stability and detectability in urinary exosomes are favorable (Han et al., 2023). Other miRNAs such as miR-377, miR-29a, and miR-126 have also been explored for their value in early diagnosis (Yun et al., 2026; Mansouri et al., 2022).

lncRNAs are ncRNAs longer than 200 nucleotides and play critical roles in kidney diseases. In DKD, their aberrant expression can impact renal cell function by acting as miRNA sponges, recruiting modifying enzymes, and more (Ignarski et al., 2019). For example, lncRNA 153 is significantly downregulated in both renal tissue and serum of DKD patients, with its expression correlated with podocyte injury and disease severity (Yu et al., 2025). IFNG-AS1 is significantly upregulated in DKD patients and is associated with inflammatory responses and renal injury, making it a “pro-inflammatory biomarker.” In contrast, TH2LCRR is notably downregulated, serving as an ideal “protective biomarker.” (Hosseini et al., 2025a) lncRNAs SNHG1 and CRNDE participate in regulating the Th17/Treg cell balance, contributing to the identification of early DKD transition (Hosseini et al., 2025b). Serum lnc458 is elevated in DKD patients and positively correlates with the severity of proteinuria (Yang et al., 2025). These findings all suggest the potential of lncRNAs as diagnostic biomarkers, though further validation is needed for clinical translation.

circRNAs have a circular structure, exhibit high stability, are enriched in blood and urine, and participate in the DKD process by acting as “molecular sponges,” among other mechanisms (Benitez et al., 2024). For example, Circ-0000953 is significantly reduced in the renal tissue of both DKD patients and mouse models; its expression correlates with urinary microalbumin, serum creatinine, and eGFR, and changes can occur before obvious kidney injury symptoms develop, indicating potential for early screening (Liu et al., 2024a). In addition, circ_0003928 and circ_0068087 have also been reported to be involved in the onset and progression of DKD (Liu et al., 2022, 2024b).

In summary, from genetic susceptibility markers to environmentally regulated epigenetic markers (especially various ncRNAs), genomics provides a wealth of information and tools for the early identification, risk stratification, and mechanistic understanding of DKD. Nevertheless, genomic markers alone offer static risk stratification without temporal sensitivity, and epigenetic markers are context-dependent and reversible; thus, their integration with functional omics layers is essential for robust early detection.

### 2.3 Construction of composite diagnostic models

**Fig. 1:**
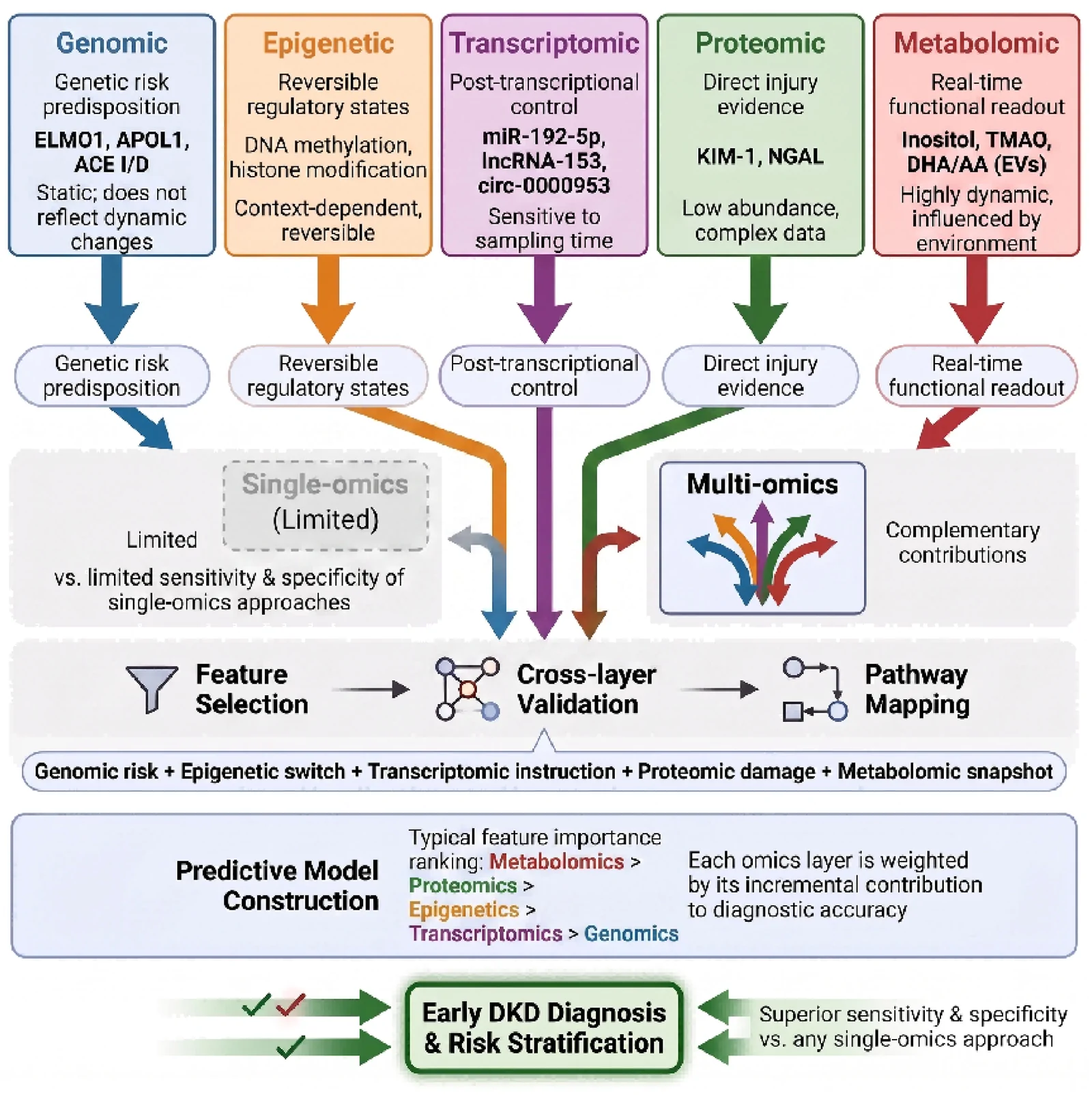
**Integrated multi-omics framework for early diagnosis and risk stratification of diabetic kidney disease.** This schematic illustrates the complementary contributions and inherent limitations of five omics layers in DKD. Genomics provides stable, germline-encoded risk predisposition (e.g., ELMO1, APOL1, ACE I/D); epigenetics captures reversible and context-dependent regulatory states (e.g., DNA methylation, histone modifications); transcriptomics reflects post-transcriptional control networks (e.g., miR-192-5p, lncRNA-153, circ-0000953); proteomics offers direct evidence of tissue injury (e.g., KIM-1, NGAL); and metabolomics delivers real-time functional readouts of systemic metabolism (e.g., inositol, TMAO, DHA/AA in EVs). In contrast to single-omics approaches, which suffer from limited sensitivity and specificity, this multi-omics integration employs feature selection, cross-layer validation, and pathway mapping to fuse heterogeneous data. Machine learning classifiers subsequently weight each layer by its incremental contribution to diagnostic accuracy. Within the current predictive model, the typical feature importance ranking was metabolomics > proteomics > epigenetics > transcriptomics > genomics, thereby enabling superior early DKD detection and risk stratification compared with any single-omics strategy. **Abbreviations:** DKD, diabetic kidney disease; EVs, extracellular vesicles; TMAO, trimethylamine N-oxide; DHA/AA, docosahexaenoic acid/arachidonic acid; KIM-1, kidney injury molecule-1; NGAL, neutrophil gelatinase-associated lipocalin; ACE I/D, angiotensin-converting enzyme insertion/deletion.

A single biomarker often fails to reflect complex DKD pathological processes, showing limited sensitivity and specificity. Integrating multi-omics data with machine learning to build composite diagnostic models is a promising approach. Fig. 1 illustrates the conceptual framework of multi-omics integration for early DKD diagnosis. At the biomarker level, five distinct omics layers each contribute unique, complementary diagnostic dimensions: genomics provides static risk stratification (e.g., ELMO1, APOL1, ACE I/D); epigenetics offers dynamic, reversible regulatory signatures (e.g., DNA methylation, histone modifications); transcriptomics captures post-transcriptional control networks (e.g., miR-192-5p, lncRNA-153, circ-0000953); proteomics reflects direct tissue injury evidence (e.g., KIM-1, NGAL); and metabolomics delivers real-time functional phenotypes (e.g., inositol, TMAO, DHA/AA in EVs). Nevertheless, each layer possesses inherent limitations when used in isolation—genomic markers are static and lack temporal sensitivity; epigenetic markers are context-dependent and reversible; transcriptomic markers lack direct phenotypic specificity; proteomic markers often emerge only after substantial structural damage; and metabolomic markers are susceptible to external confounders such as diet and drugs. Multi-omics integration overcomes these individual limitations by fusing their complementary contributions through feature selection, cross-layer validation, and pathway mapping, ultimately constructing predictive models that outperform single-omics approaches in sensitivity and specificity (Fig. 1).

Multi-omics technologies can systematically integrate diverse information such as genomics and metabolomics, comprehensively analyzing disease mechanisms—such as identifying differentially expressed genes (like FN1, ALDH2) and associated pathway abnormalities (Lin et al., 2025; Liu et al., 2025; Luo et al., 2024).For instance, combining urinary metabolomics and peptidomics identified stepwise-regulated metabolites and peptides (e.g., UMOD, SERPINA1) that improve early DKD diagnosis (Jiang et al., 2023). Machine learning algorithms efficiently process high-dimensional data and select feature biomarkers, overcoming reliance on single indicators.

Recent studies utilized routine blood and biochemical parameters (e.g., TyG index, creatinine) to construct logistic regression models for early DKD screening (Yong et al., 2026), and integrated inflammatory–metabolic indices (e.g., UHR, NHR, SII) into XGBoost models with robust performance (Liu et al., 2026). Importantly, SHAP (Shapley Additive Explanations) analysis—based on cooperative game theory—quantitatively decomposes model predictions and assigns a contribution value to each biomarker, addressing the “black box” limitation of machine learning. As shown in Fig. 1, each omics layer is weighted by its incremental contribution to diagnostic accuracy, with a typical feature importance ranking of Metabolomics > Proteomics > Epigenetics > Transcriptomics > Genomics. For instance, SHAP analysis ranked eGFR, albumin, and C3 as top contributors to DKD prognosis, while peptidomic and metabolomic markers provide complementary, quantifiable information (Qian et al., 2026). Such interpretable frameworks enable precise quantification of each biomarker's diagnostic contribution, offering a strategy for translating multi-omics biomarkers into clinically actionable models.

**Fig. 2:**
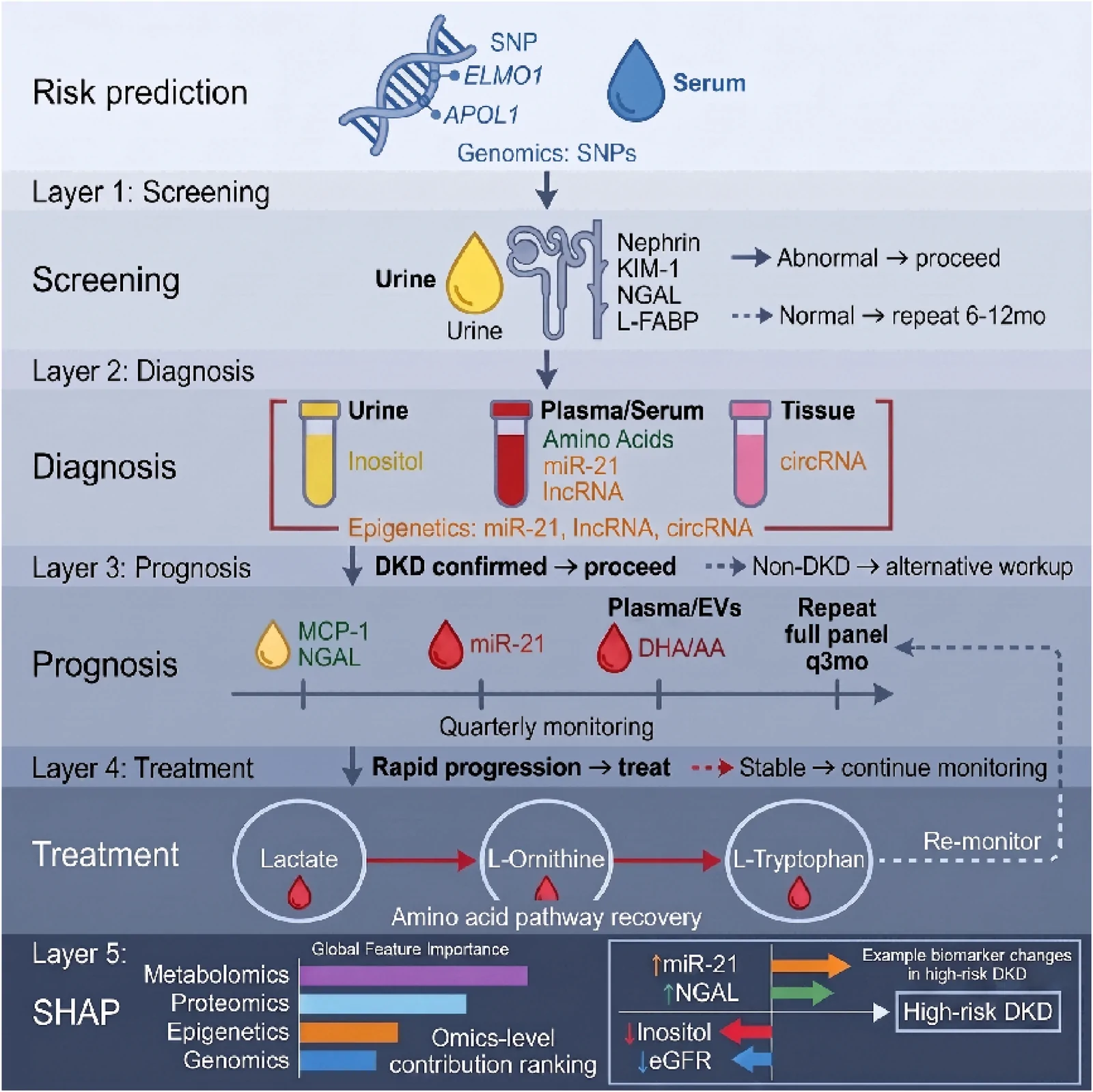
**Multi-omics-based clinical workflow and interpretable risk stratification for diabetic kidney disease.** This schematic presents a stepwise diagnostic and prognostic algorithm (Layer 1: Screening to Layer 5: Model Interpretability) integrating genomic, epigenomic, metabolomic, and proteomic biomarkers from serum, urine, and tissue specimens. Layer 1 (Screening) identifies initial genomic risk via SNPs (e.g., ELMO1, APOL1) and urinary damage markers (NGAL, L-FABP); abnormal results trigger progression to Layer 2 (Diagnosis), which confirms DKD via urinary/plasma miRNAs, circRNAs, and lncRNAs. Confirmed cases proceed to Layer 3 (Prognosis), wherein serial monitoring of plasma/EVs markers (MCP-1, miR-21, DHA/AA) defines quarterly (q3mo) follow-up. Layer 4 (Treatment) evaluates metabolic recovery through amino acid pathway restoration (lactate, L-ornithine, L-tryptophan) to guide therapeutic decisions. Layer 5 employs two complementary interpretability tools: (a) a global feature importance bar plot ranking omics-layer contributions (metabolomics > proteomics > epigenetics > genomics) to the predictive model; and (b) a SHAP force plot providing patient-level explanation, where deviations in key biomarkers (↑miR-21, ↑NGAL, ↓inositol, ↓eGFR) drive individual risk classification toward “High-risk DKD.” **Abbreviations**: DKD, diabetic kidney disease; EVs, extracellular vesicles; L-FABP, liver-type fatty acid-binding protein; MCP-1, monocyte chemoattractant protein-1; miRNA, microRNA; NGAL, neutrophil gelatinase-associated lipocalin; SHAP, SHapley Additive exPlanations; SNP, single nucleotide polymorphism. **Symbol and Line Legend:** Sample icons: Yellow = urine; red = plasma/serum; pink = tissue biopsy. Solid arrows: Defined workflow progression; Dashed arrows:Alternative workup or repeat monitoring pathways. Colored arrows: Red (↑) denotes biomarkers whose elevated levels push prediction toward high-risk; Blue (↓) denotes markers whose reduced levels are associated with adverse renal outcomes (e.g., ↓inositol, ↓eGFR). Circular nodes & timeline: Key metabolites in the amino acid recovery pathway; quarterly (3-month) monitoring intervals for dynamic markers.

Building upon the multi-omics integration framework illustrated in Fig. 1, where heterogeneous biomarker contributions are systematically fused through machine learning, we translated this conceptual model into a clinically actionable stratified diagnostic algorithm (Fig. 2). Based on the characteristics of the various biomarkers described above, we propose a stratified integrated diagnostic algorithm that systematically incorporates serum, urine, and genomic biomarkers according to the clinical application scenarios. This algorithm is centered on the four-dimensional complementarity of “genetic–structural–functional–mechanistic” markers and encompasses five progressive tiers: the risk prediction tier (Layer 0) incorporates SNPs (such as ELMO1 and APOL1) and epigenetic markers to assess individual DKD genetic susceptibility at the initial diagnosis of diabetes; the primary screening tier (Layer 1) combines urinary podocyte markers (Nephrin, Podocalyxin) with tubular markers (KIM-1, L-FABP, and NGAL) to identify structural injury even during normoalbuminuric stages; the diagnostic tier (Layer 2) introduces urinary metabolomic features (such as myo-inositol) and plasma amino acid profiles, supplemented by miRNA (miR-21 and miR-192-5p) and lncRNA (IFNG-AS1 and TH2LCRR) to aid in differentiating DKD from non-diabetic kidney disease; the prognostic monitoring tier (Layer 3) dynamically tracks urinary MCP-1/NGAL, plasma EVs metabolites (DHA and AA), and circRNA (circ-0000953) changes, in combination with eGFR slope and UACR trends to assess risk of progression; the therapeutic decision-making tier (Layer 4) uses serum metabolites (lactate, L-ornithine, and L-tryptophan) to monitor recovery of amino acid pathways, guiding efficacy evaluation and regimen adjustment for drugs such as RAS inhibitors and SGLT2i. This stratified framework not only addresses the lack of specificity inherent in single biomarkers (see Table 1 for details) but also clarifies the complementary roles of multi-omics biomarkers at different clinical nodes.

The construction of such models usually follows a core technical process that mirrors the integration pipeline depicted in Fig. 1. First, multi-omics data and traditional clinical indicators (such as UACR, eGFR) are collected from samples like plasma and urine, and undergo rigorous quality control and standardization to ensure comparability. Second, core biomarker panels are identified using algorithms such as LASSO regression and random forest, which correspond to the feature selection stage of Fig. 1. Third, SHAP analysis is applied to quantify the contribution of each omics biomarker to model prediction, thereby identifying key global drivers (e.g., metabolomic biomarkers often contribute most) as well as localized individual explanations (e.g., the interaction effect between miR-21 and myo-inositol in specific patients), thus revealing synergistic and redundant relationships among biomarkers—consistent with the cross-layer validation and pathway mapping stages of Fig. 1. Next, these multi-omics biomarkers are integrated with traditional metrics to build predictive models. Finally, the performance indices of the models, such as sensitivity, specificity, and area under the curve (AUC), are evaluated in independent cohorts and optimized through methods such as cross-validation to ensure generalizability.

Ultimately, these biomarkers are integrated with traditional clinical indicators (UACR, eGFR) to construct interpretable stratified prediction models, with generalizability further optimized via cross-validation and external validation. Existing research has demonstrated the effectiveness of this approach—for example, by integrating multi-omics data from the GEO database, key biomarkers such as AGR2 and CCR2 have been identified using WGCNA and LASSO regression, with external validation confirming satisfactory diagnostic performance (Wang et al., 2024; Liu et al., 2023). Future research could further incorporate SHAP analysis, not only improving the predictive accuracy of the model but also enhancing its clinical interpretability, thus transforming the “black box” model into a decision support tool that physicians can understand.

In summary, multi-omics integration can characterize disease heterogeneity at the molecular level, overcoming the limitations of traditional staging, while machine learning enhances the objectivity of biomarker selection and the generalizability of models. The combination of the two not only shows promise for addressing clinical challenges such as delayed early diagnosis and ambiguous subtyping of DKD, but also offers new avenues for precision medicine. Future research should focus on intelligently integrating information from multiple sources to build scenario-based precision diagnostic pathways.

## Special note

As an important foundation for the viewpoints of this review, Zhang Zhuxian, as the first author, published a relevant study (Zhang et al., 2021): ZHANG Z, HE P, ZHOU C et al. Association of estimated glomerular filtration rate from serum creatinine and cystatin C with new-onset diabetes: a nationwide cohort study in China [J/OL]. Acta Diabetologica, 2021, 58 (9): 1269-1276. https://doi.org/10.1007/s00592-021-01719-5. This study explored the early diagnosis of diabetic nephropathy. This review further expands upon the summary and reflection on early diagnostic markers for diabetes.

## Consent for publication

Written informed consent for publication was obtained from the participant.

## Author contributions

Haimin Wang: Supervision, validation, and visualization.

Zihan He: Project administration and software.

Jiao Meng: Formal analysis, methodology, and writing – review and editing.

Wei Zhang: Funding acquisition and writing – original draft.

Zhuxian Zhang: Conceptualization.

All authors contributed to the article and approved the final submitted version.

## Submission and originality

The work described is original, has not been published previously, and is not under consideration for publication elsewhere. All authors have read and approved the manuscript for submission to the Current Research in Physiology. The corresponding author (Meng Jiao) is responsible for all communication during the submission and review process.

## Funding

This study was supported by the Yunnan Provincial Department of Science and Technology Program (202501AY070001-204) and an internal research project of Qujing First People's Hospital [Grant No. 2022YJKTY14]. The funders had no role in study design, data collection and analysis, decision to publish, or preparation of the manuscript.

## Declaration of competing interests

The authors declare that they have no known competing financial interests or personal relationships that could have appeared to influence the work reported in this paper.

## Data Availability

No data was used for the research described in the article.
